# The Locust

**Published:** 1834

**Authors:** N. M. Hentz

**Affiliations:** Cincinnati


*Cincinnati, June,* 1834.

### VII.—The Locust.

The locust, the devastations of which are recorded in the Scriptures, is a kind of grasshopper, belonging to the order

Orthoptera, characterized by the presence of Maxillœ and mandibles or jaws for mastication, and of wings which are folded longitudinally. There are several species of locusts, (Gryllus Linn.) which were a plague as well as food to man in Asia and Africa. They now, as of old, elevate themselves high in the atmosphere, and are freqently borne by currents of air to Europe, where, in a few hours, the destruction produced by their appetite for leaves, is followed by the contamination of the air, occasioned by the myriads of their dead bodies heaped on the desolated soil. At this day they are eaten by man, as well as in the time of St. John. A kind of meal is made of them after being dried, and they are also pickled in such quantities as to render them an article of commerce.

The locust, so called, which has appeared in this country this season, belongs to the order Hemiptera, in which the bed-bug is found, and which is characterised by having neither mandibles nor maxillæ or jaws, but a *Rostrum;* and consequently the insects of the order are incapable of eating and committing the same kind of ravages as the Eastern fly. The name of locust, is, therefore, erroneous, and, as in many other cases, has given rise to many errors, which this paper is intended to correct. The true appellation of the insect is Cicada Linn. (Tettigonia Fab.) *Septendecim.* The Greeks called it *tettix* and *achetes.* Hence Fabricius took his name; but the name Cicada, the Latin word, should be retained. It occurs in Pliny, and in the following lines of Virgil, we find one of the characters of the insect truly and elegantly described.

At mecum *raucis* tua dum vestigia lustro,
*Sole sub ardenti resonant arbusta cicadis.*

The *cicadœ* suck the sap of the trees in their three states of larva, puppa, and fly. The females, by means of the ovipositor or sword which is contained in their abdomen, deposit their eggs under the bark of the branches which they saw longitudinally, from five to eight eggs are found in each incision, and a female can lay five or six hundred. White *larvœ* or worms are hatched from these eggs which do not exceed the size of a flea. They descend along the bark of the branches and stem and bury themselves in the ground, where they suck the roots of the trees at the depth of one or two feet; by the end of the first season, or the beginning of the next, they are transformed into the *puppa* state, having already the rudiments of wings, and fore feet which are strong, designed to burrow, and to climb the trees when they ultimately come out for their last transformation.

These remarks apply to the many species belonging to the genus Cicada. We have here several which appear every year, one of which is common and much larger, making a very similar noise. As to the seventeen years locust, as it is commonly called, little is known concerning the long period during which it remains under ground. The writer of this article, never saw one of those insects, but when they appear in a vast body; but he has seen the myriads in New-York, in North Carolina and in Ohio, in the space of nine years. Dr. J. Locke of this place, showed him the puppæ of the seventeen years locust, which were found near the surface of the ground, more than six weeks before the perfect insect appeared, and there seemed to be a general anticipation that this was to be the year of their periodical visit; but the writer is not yet convinced, by any printed account of their appearance, that seventeen, or eight, or seven are the number of years which are necessary to complete the subterranean existence. It is evident that they are not a migrating insect like the *Locusta* of the East, and that the swarm which we have now, cannot be the offspring of the one, for instance, which appeared four years ago in North Carolina; but did the locusts really show themselves last in 1817, in this place? When that fact is proved by print or notes made at the time, the writer, who has no kind of objection to believe it, will be at once convinced. The havock committed by the true locust of Asia and Africa, has led many to believe that the leaves of many trees, which were injured by the late frost, were eaten by our obstreporous visitors. Let them examine the proboscis of these insects, and they will be convinced that they cannot devour vegetables much better than the bed-bug could grind corn or hickory-nuts. The damage done to the young twigs by the insertion of the eggs, is not very material, and is the only harm which can be produced by them. As to the stench which might be produced by their dead bodies, other insects, birds and hogs are more than sufficient to preserve the purity of our atmosphere in that respect. N. M. HENTZ.

